# Identification of key pathways and hub genes in the myogenic differentiation of pluripotent stem cell: a bioinformatics and experimental study

**DOI:** 10.1186/s13018-020-01979-x

**Published:** 2021-01-04

**Authors:** Wenyong Fei, Mingsheng Liu, Yao Zhang, Shichao Cao, Xuanqi Wang, Bin Xie, Jingcheng Wang

**Affiliations:** 1 Sports Medicine Department, Northern Jiangsu People’s Hospital, Clinical Medical College, Yangzhou University, 98# Nantong xi Road, Yangzhou, 225001 China; 2 Dalian Medical University, Dalian, 116044 Dalian China

**Keywords:** Stem cells, Muscular regeneration, MyoD, Actin, Bioinformatics analysis

## Abstract

**Background:**

The regeneration of muscle cells from stem cells is an intricate process, and various genes are included in the process such as myoD, mf5, mf6, etc. The key genes and pathways in the differentiating stages are various. Therefore, the differential expression of key genes after 4 weeks of differentiation were investigated in our study.

**Method:**

Three published gene expression profiles, GSE131125, GSE148994, and GSE149055, about the comparisons of pluripotent stem cells to differentiated cells after 4 weeks were obtained from the Gene Expression Omnibus (GEO) database. Common differentially expressed genes (DEGs) were obtained for further analysis such as protein-protein interaction (PPI) network, Gene Ontology (GO), Kyoto Encyclopedia of Genes and Genomes (KEGG), and GSEA analysis. After hub genes and key pathways were obtained, we manipulated in vitro cell research for substantiation such as immunohistochemical staining and semi-quantitative analysis and quantitative real-time PCR.

**Results:**

A total of 824 DEGs including 350 upregulated genes and 474 downregulated genes were identified in the three GSEs. Nineteen hub genes were identified from the PPI network. The GO and KEGG pathway analyses confirmed that myogenic differentiation at 4 weeks was strongly associated with pathway in cancer, PI3K pathway, actin cytoskeleton regulation and metabolic pathway, biosynthesis of antibodies, and cell cycle. GSEA analysis indicated the differentiated cells were enriched in muscle cell development and myogenesis. Meanwhile, the core genes in each pathway were identified from the GSEA analysis. The in vitro cell research revealed that actin cytoskeleton and myoD were upregulated after 4-week differentiation.

**Conclusions:**

The research revealed the potential hub genes and key pathways after 4-week differentiation of stem cells which contribute to further study about the molecular mechanism of myogenesis regeneration, paving a way for more accurate treatment for muscle dysfunction.

**Supplementary Information:**

The online version contains supplementary material available at 10.1186/s13018-020-01979-x.

## Introduction

As the human population is aging, muscle dysfunction has been an interrupting issue in clinical research [1–4]. A series of diseases were correlated to the atrophy of skeletal muscles and leading to dysfunction of muscular organs such as Duchenne muscular dystrophy (DMD), degenerative rotator cuff tear, etc. [5–7]. Stem cells are promising cells that have the potency of multi-directional differentiation and proliferation and are widely expected to be used in the field of tissue repair and regeneration [8, 9]. In the muscle regeneration field, stem cells also showed vigorous potency [10].

Various researches have devoted to verifying the mechanism of myoblast differentiation. In myogenic differentiation, as a multistage process, there stay several regulating factors such as Myf5, Myf6, myoD, and myog [11–13]. Meanwhile, researches have shown that different factors were correlated with different stages at the myogenic process such as myoD at the late stage and myf5 at the early stage [14–16]. What is more, couples of pathways were verified to be correlated to myogenic differentiation such as PI3K-MAPK, p38, p53, and actin pathway [14–17], but few researches have shown the pathway variation at differentiation stages. To upregulate the differentiation efficacy and contribute to the repair of degenerated muscular tissues, it is especially important to clarify the differentiation mechanism at the genetic level.

With the widespread use and development of high-throughput sequencing, bioinformatics analysis showed a great advantage for determining the myogenic differentiation mechanism of stem cells at the genetic level. However, no study was designed to integrate the myogenic differentiation datasets in GEO. In the present study, we integrated 3 datasets in GEO comparing human pluri-potential stem cells and myogenic stem cells. Bioinformatics analysis was used to explore the molecular mechanism of the pathogenesis in myogenic differentiation of stem cells.

## Materials and methods

### Microarray data obtained

Three gene expression profiles, GSE131125 (GPL 20844, SurePrint G3 Human GE v3 8x60K Microarray 039494), GSE148994, and GSE149055 (GPL16686, Affymetrix Human Gene 2.0 ST Array), were obtained from the GEO database. Both the GSE149055 and GSE148994 contained 6 samples, of which 3 were undifferentiated stem cells and 3 were differentiated cells after 30-day differentiation. GSE133125 contained 24 samples which include different time points of the differentiation. We choose the 3 undifferentiated stem cells and 3 differentiated for 25 days into our analysis.

### Identification of differently expressed genes (DEGs)

The downloaded platform files were matched to the gene expression profiles by the “VLOOKUP” function of Excel 2010. Gene differential analysis was determined to summarize the differentially expressed genes (DEGs). The DEG threshold of our study was |logFC| > 1 and adj.*P* value < 0.01. Heatmaps of DEGs from 3 groups were generated by GraphPad 8.0.2. Online tool Venn, version 2.1(bioinformatics.psb.ugent.be/webtools/Venn; version 2.1), was used to determine the common DEGs among the three profiles.

### Protein–protein interaction (PPI) network construction and module selection

Search Tool for the Retrieval Interacting Genes (STRING) database was used to construct the network of differentially expressed genes and proteins, and Molecular Complex Detection (MCODE; version 1.31) in the Cytoscape (version 3.8.0) was used to analyze modules in the network.

### GO and pathway enrichment analysis construction

Both the GO and KEGG analyses were applied under the online program Database for Annotation, Visualization and Integrated Discovery (DAVID, version 6.8) whose subgroup of functional annotation tools can help the researchers to understand the biological meanings about the selected genes. Gene Set Enrichment Analysis (version 4.0.3) was used to verify whether DEGs showed statistical significance in one phenotype or pathway based on the expression profiles.

### Isolation and cultivation of ADSCs

An 8-week-old New Zealand white rabbit (Animal Experiment Center of Jiangsu University) weighing 2.0 kg was sacrificed under the guidelines of the Institutional Animal Care and Use Committee of Jiangsu University, China. The rabbit was kept and fed in a single cage in housing conditions. Housing was controlled in temperature (25 °C), humidity (40–60%), and light (12 h, light–dark cycle). Animals were observed for 1 week before surgery to confirm that they were healthy and disease-free.

0.6% sodium pentobarbital (4 mg/kg) was injected into the rabbits’ ear veins for general anesthesia. Then, 2% lidocaine hydrochloride was injected into the planned skin incision to enhance the effect of anesthesia. Prior to placing the animals in a laminar flow chamber, the hair was clipped at the abdominal area. An incision was made along with the linea alba to expose the peritoneum, and the inguinal fat was removed.

The adipose tissue was washed three times with phosphate-buffered saline (PBS) to remove red blood cells. The collected adipose tissue was cut into small pieces and transferred into one a 20-mL centrifuge tube, and an equal volume of a 0.25% trypsin (Gibco, USA) and 0.1% type I collagenase (Sigma, USA) mixture was added. The tissue was incubated on shaking tables at 37 °C with constant agitation for approximately 15 min. Afterwards, the liquid was separated into three layers: the upper layer contained yellow oily lipocytes, the intermediate layer contained adipose tissue, and the bottom layer contained mononuclear cells. The bottom layer was extracted and transferred into a centrifuge tube containing 15% fetal bovine serum (FBS, Gibco, USA) and high-glucose DMEM (Sigma, USA). The remaining stromal fractions were treated with 3 mL red blood cell lysis buffer (Sigma, USA) for 10 min at room temperature, filtered through a 100-mm nylon mesh, and centrifuged at 1200×*g* for 10 min; then, the supernatant was removed. The cell pellets were then suspended in high-glucose DMEM containing 15% FBS (Gibco Company, St. Louis, MO, USA), 100 U/mL penicillin, and 100 mg/mL streptomycin (Gibco Company, St. Louis, MO, USA). The cells were cultured at 37 °C and 5.0% CO_2_ in a humidified incubator, with full media replacement every 3 days. When the cells reached 80% confluence, they were digested with a mixture of 0.25% trypsin and 0.04% EDTA (Shanghai Reagent, China) and passaged for later use.

### Flow cytometry (FCM) analysis of ADSCs

Passage-3 adherent cells were treated with 0.25% trypsin (Gibco, USA) and washed twice with PBS. The cells were incubated with rabbit anti-CD45 and anti-CD90 antibodies (Invitrogen, USA, and Gibco, USA) overnight at 4 °C. Unbound antibodies were removed by washing three times with PBS. After washing, the cells were incubated for 45 min at room temperature in the dark with Cy3-labeled secondary anti-goat/anti-rabbit antibody and resuspended in PBS for FACS analysis. At least 1 × 10^6^ cells per sample were analyzed with a flow cytometer (BD FACSVerse, USA). CELLQuest software was used for the analysis.

### Assessment of cell viability by tetrazolium (MTT) method

The cell viability was quantitatively determined by the tetrazolium (MTT) method. MTT is a yellow tetrazolium dye which responds to metabolic activity. The reductases in living cells reduce MTT from a pale-yellow compound to dark-blue formazan crystals. The passage-3 ADSCs were digested and diluted, and the mixture was transferred to a 96-well culture plate (Thermo Scientific, USA) at 1 × 10^5^ cells per well. 5-Aza was then added to each well at concentrations of 0, 10, 20, 30, and 40 μmol/L. Then, at 24 h, 48 h, and 72 h after induction, the absorbance which represents cell viability was tested in each group. Firstly, the supernatant was removed. Then, 200 μL of dimethyl sulfoxide (DMSO; Merck, Germany) was added to each well to dissolve the blue substance. Finally, the absorbance (OD) at 570 nm was read using a microplate reader (Biotek, USA).

### Induction of differentiation of ADSCs by 5-azacytidine (5-Aza)

The passage-3 ADSCs were digested by a mixture of trypsin and EDTA and diluted to single-cell suspension of 10^4^ cells/mL and then seeded into cell culture flasks. Groups A, B, and C were induced by 0, 10, and 20 μmol/L 5-Aza (Sigma, USA), respectively, for 24 h and washed with D-Hank’s balanced salt solution (HBSS, Gibco Company, St. Louis, MO, USA). Then, the medium was replaced with low-glucose DMEM containing 10% FBS. The cells in each group were incubated at 37 °C with 5% CO_2_ in a conventional incubator. The medium was replaced with fresh DMEM and FBS every 3 days until the test begins after 25-day cultivation.

### Immunohistochemical staining and semi-quantitative analysis

We determined the KEGG pathway of actin cytoskeleton about the expression of actin by immunohistochemical staining. The cells were digested and diluted 25 days after induction and added 150 μL 4% paraformaldehyde fixative to every slide and left them undisturbed for 30 min before adding 150 μL 0.1% Triton X-100 microplate reader (Biotek, USA). Primary antibody α-SMA (1:200) (Proteintech, USA), secondary antibody (1:200) (Proteintech, USA), and Hoechst33258 stain (C1011 Beyotine, China) were added to each slide in a dark environment at room temperature. Finally, we observed the cells under a fluorescence microscope (Leica, Germany), photographed, and stored them. ImageJ (Rawak Software, Germany) software was used for photography and Prism Demo software for data statistics (GraphPad Software, USA).

### Quantitative real-time PCR

Total RNA was extracted from the ADSCs after induction of 25 days using Trizol lysate (Invitrogen). The schizolytic cells were then transferred into another tube without RNA enzymes, and 200 μL pre-cooling chloroform (Sigma Centrifuge, Germany) was added per milliliter of Trizol. The centrifugation yielded RNA sediments that were preserved in a − 20 °C surrounding for 30 min. The sediments were washed with 75% ethyl alcohol and centrifuged for 5 min, and the supernatant was discarded after washing and centrifuging the sediments twice. The reverse transcription system was prepared using a reverse transcription kit (Thermo Scientific, USA) according to instructions provided in the protocol of the kit.

### Statistical analysis

Statistical analysis was performed on Graphpad 8.0.2 and R 4.0.0. Expressed data were shown as mean ± SD. Student’s *t* test was used to evaluate the statistical significance of the different 3 groups. *P* value less than 0.05 was considered significant.

## Results

### Identification of DEGs

**Fig.1:**
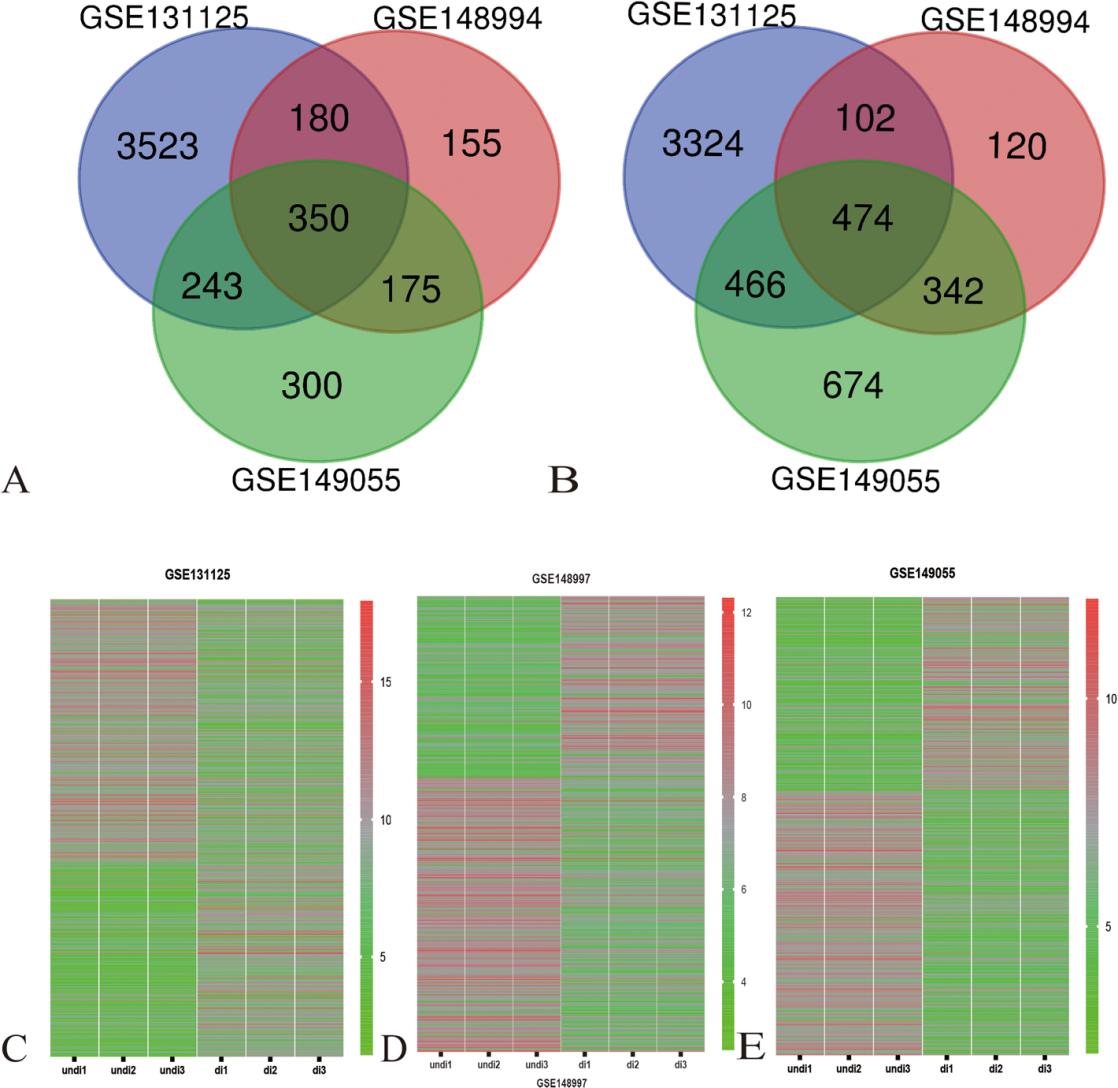
**a** Venn diagram of upregulated DEGs across different profiles. **b** Venn diagram of upregulated DEGs across different profiles. **c** Heat map of DEGs in GSE131125. **d** Heat map of DEGs in GSE148994. **e** Heat map of DEGs in GSE140955

The three datasets were standardized, and the results are shown in Fig. 1. The threshold of DEG determination was that |LOG(FC)| lower than 1 and adj.*P* value lower than 0.01. From the GSE131125 database, there were 5051 upregulated and 5199 downregulated DEGs. Meanwhile, 864 upregulated and 1038 downregulated DEGs were calculated from GSE148994. As for GSE149055, there were 1068 upregulated and 3913 downregulated DEGs. The heat map of DEGs in each dataset is shown in Fig. 1c–e. The DEGs in each group were mixed by the Venn plot. From the Venn plot shown in Fig. 1a and b, there are 824 common DEGs among the three subgroups, of which 350 were upregulated DEGs and 474 were downregulated.

### Protein–protein interaction (PPI) network construction and sub-modules

**Fig. 2:**
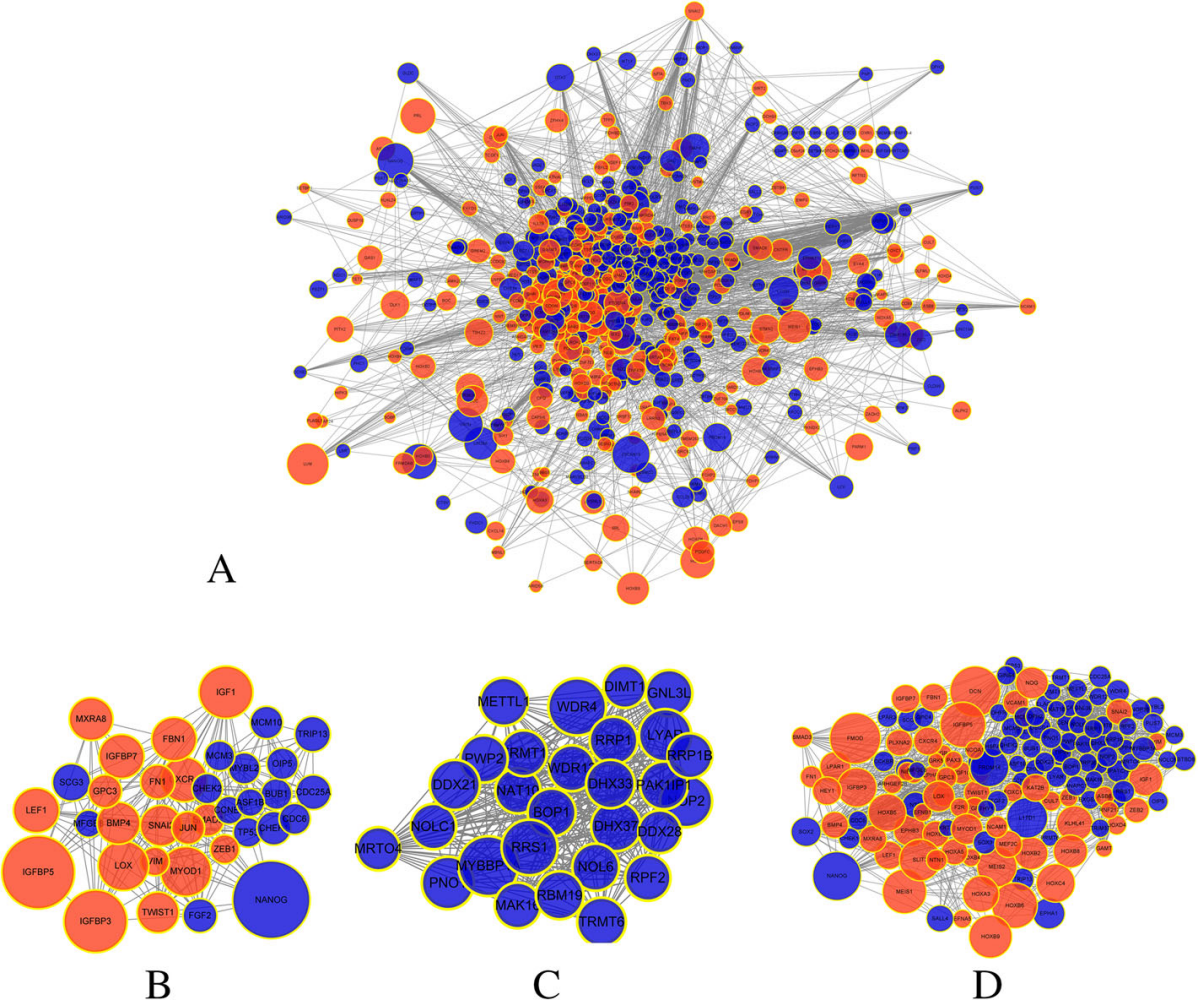
**a** Potein–protein interaction (PPI) network construction. A total of 824 DEGs were identified. **b**–**d** The significant top 3 modules in the PPI network. Genes with blue represent downregulated genes. The size of each gene was based on the interaction analysis that bigger size indicates more interactions

Eight hundred twenty-four notes and 3200 edges consist the full network shown in Fig. 2a. Meanwhile, with the aid of the MCOD app, the top 3 modules are selected and shown in Fig. 2b–d with 28 notes and 349 edges in module 1, 36 notes and 237 edges in module 2, and 47 notes and 176 edges in modules 3. From the MCOD function, 19 hub genes were selected: ASXL1, BOC, CENPH, DIMT1, ESRP1, GLDC, HOXD3, IGFBP5, JUN, MGST1, MRPS34, MSTN, MYOD1, MYOG, NBAS, PLS1, POLR3G, RNF144B, and UST.

### GO and pathway enrichment analysis from the DEGs

**Fig. 3:**
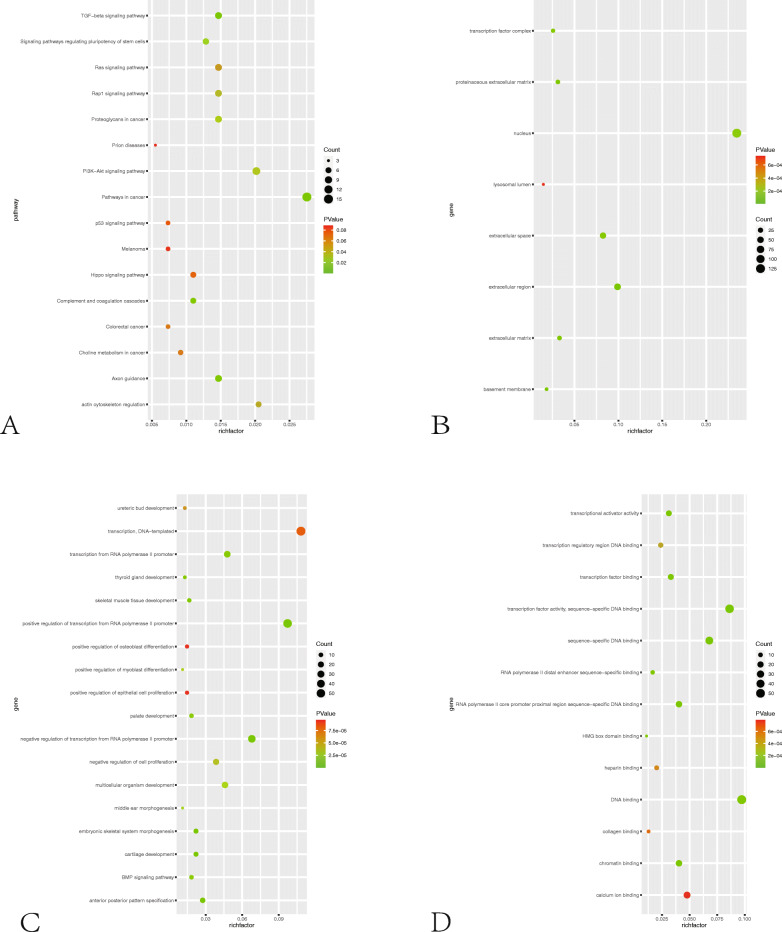
**a** Bubble diagram of KEGG pathway analysis and GO analysis of upregulated genes. (A) KEGG analysis of upregulated genes. (B) CC functional classification terms of upregulated genes. (C) BP functional classification terms of upregulated genes. (D) MF functional classification terms of upregulated genes

**Fig. 4:**
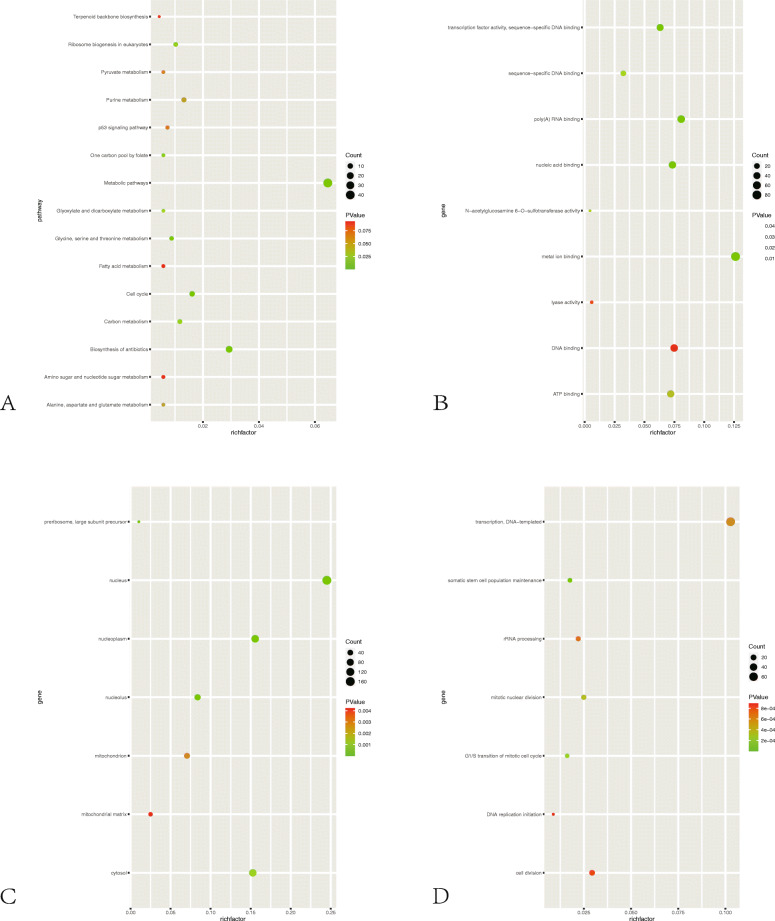
**a** Bubble diagram of KEGG pathway analysis and GO analysis of downregulated genes. (A) KEGG analysis of downregulated genes. (B) MF functional classification terms of downregulated genes. (C) BP functional classification terms of downregulated genes. (D) CC functional classification terms of downregulated genes

**Fig. 5:**
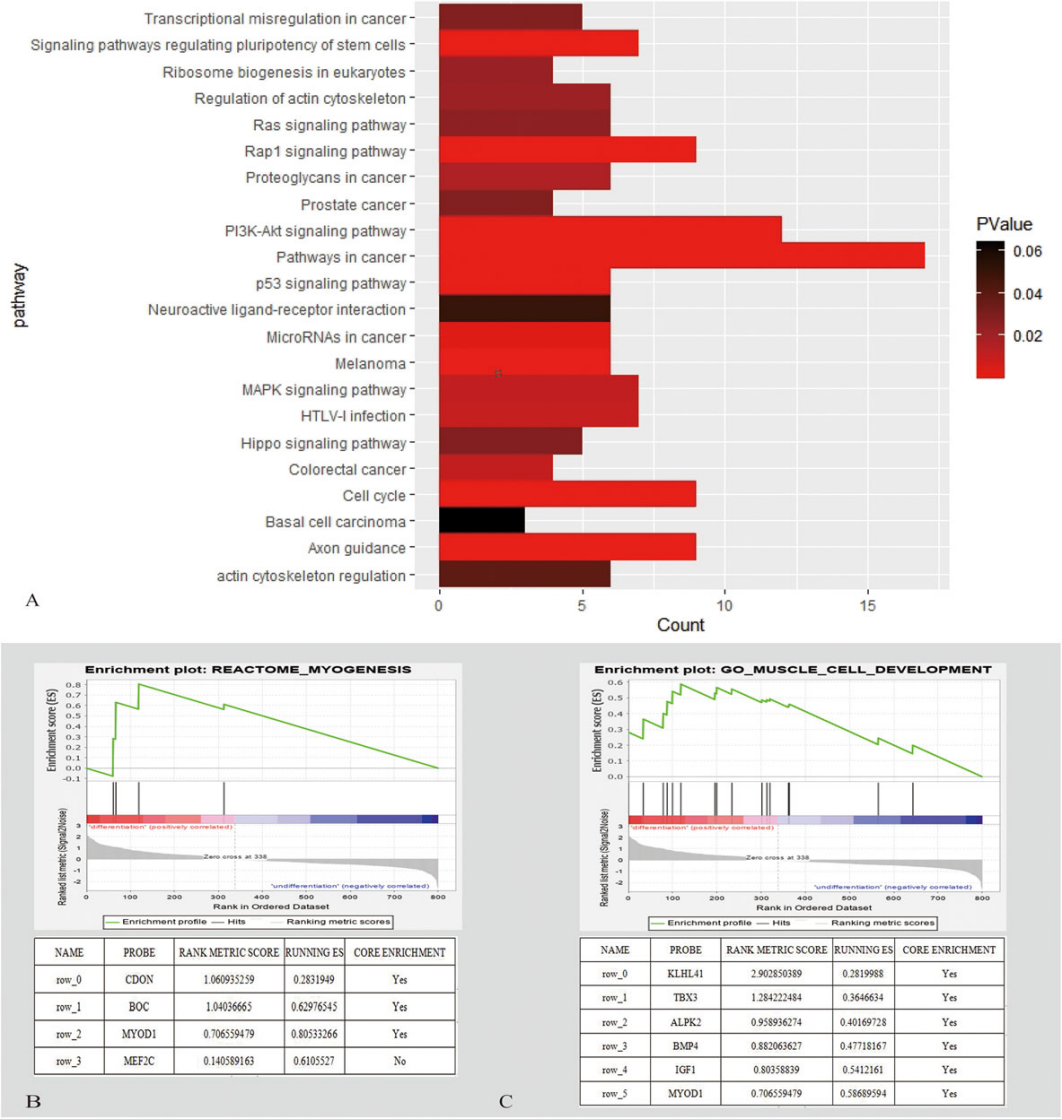
**a** KEGG analysis and GSEA analysis based on DEGs. (A) Histogram of KEGG pathway analysis of DEGs. (B) GSEA analysis of myogenesis pathway and their core genes. (C) GSEA analysis of muscle cell development and their core genes

The GO analysis was processed to determine the function distributions of common DEGs from three aspects. Figure 3a and Additional file 1: Figure 3b and 3c show upregulated DEG enrichment including KEGG pathways, molecular function (MF), biological processes (BP), and cell composition (CC). In KEGG analysis, the top 3 enriched pathways were pathway in cancer, PI3K pathway, and actin cytoskeleton regulation. DEGs were enriched in transcription functions in BP, extra-celluar communications in CC, and DNA binding in MF. Meanwhile, the downregulated DEGs shown in Fig. 4a and Additional file 1: Figure 4b and 4c are mainly distributed in the metabolic pathway, biosynthesis of antibodies, and cell cycle. In the upregulated function analysis, from BP to CC and MF, MYOD1 showed significantly differentially expressed. According to the KEGG analysis, the enriched pathway “actin cytoskeleton regulation” was on the way of myogenic differentiation. The GSEA analysis of DEGs was processed, and the results are shown in Fig. 5a (B, C). The DEGs were enriched in “myogenesis” and “muscle cell development.” MyoD1 was the “core enrichment” gene in both enriched pathways. Therefore, we set the MyoD1 as the hub gene and “actin cytoskeleton regulation” pathway as the mainly enriched functional pathway.

### The expression of ADSC and BMSC surface markers

**Fig. 6:**
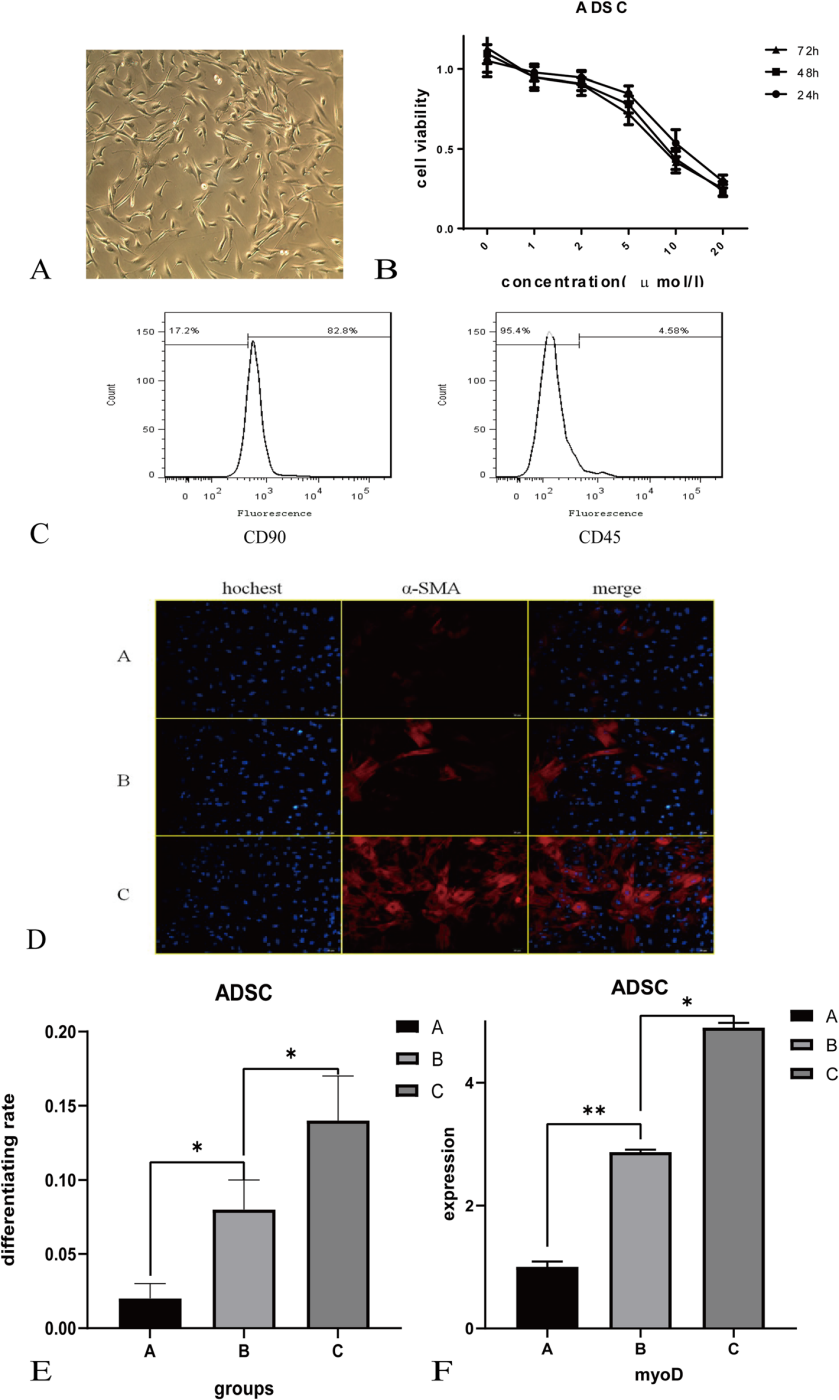
Experimental certification of the hub genes and key pathways. **a** Cellular morphology of passage-3 ADSCs. **b** MTT assessment showing the effect of different concentrations of 5-Aza on the viability of ADSCs at passage 3 after 1, 3, 5, 7, and 9 days of exposure. The *x*-axis is the time (days), and the *y*-axis is the cell viability value. **c** Flow cytometry analysis results and expression of cell surface CD markers of ADSCs at passage 3. The *x*-axis is the fluorescence intensity, and the *y*-axis is the cell number. **e** Immunofluorescence analysis of ADSCs. The results represent sarcomeric-α-actinin expression in ADSCs exposed to three myogenic concentration protocols. **e** Myogenic differentiation rates are represented by the percent expression of actin as measured by immunohistochemistry. **f** MyoD (RT-PCR) mRNA expression levels

Specific membrane markers confirmed the identity of ADSCs via flow cytometry. According to the results, the ADSC results are presented in Fig. 6c, with a strong expression of CD90 at 82.8% positive and weak expression of CD45 at 4.58% positive and the results are shown in Fig. 6c wherein the *x*-axis is the fluorescence intensity and the *y*-axis is the cell number.

### Cell viability authenticated by MTT

The MTT results are converted into Fig. 6b to show cell viabilities when different concentrations of 5-Aza induced the cells. As is shown in Fig. 6b, 5-Aza does have dependent and time-dependent toxic effects on ADSCs. As the concentration of 5-Aza increased, the absorbance was significantly decreased. The results revealed that an increased concentration of 5-Aza showed increased toxic effects on ADSCs. Meanwhile, when compared with different time points after induction, the absorbance at 48 h and 72 h were significantly decreased when compared to 24 h after induction. The results revealed that an increased induction duration of 5-Aza showed increased toxic effects on ADSCs. It can be calculated that the IC50 in ADSC groups were 9.178 μmol/L at 24 h after induction. The further induce concentration of 5-Aza was set as 0, 10, and 20 μmol/L and named as groups A, B, and C, respectively.

### Actin expression determined by immunohistochemistry

The results of the expression of actin are shown in Fig. 6d. Actin was labeled and stained red by α-SAM, and the nucleus was stained blue by Hochest, with the composed pictures showing that there were just parts of the cells expressing actin. The differentiated rate was calculated by GraphPad 8.0.2. And the rate of each group was 0.019, 0.074, and 0.116 for groups A, B, and C, respectively. The differentiation rate in groups B and C was significantly upregulated when compared to group A (*P* < 0.05).

### The content of myoD mRNA measured by RT-PCR

We further used the RT-PCR technology to detect the content of the mRNA of myoD in each group under the induction of 5-Aza. The results were recorded at 1.009, 2.391, and 4.876, respectively, in each group, of which the content in group C was significantly upregulated than that in group B (*P* < 0.05) whose content was also upregulated compare to group A with significance (*P* < 0.05).

## Discussion

Mountainous efforts have been devoted to the research of pluripotent stem cells in our nowadays research for their regenerating and repairing damaged tissue effects [18–20]. In the musculoskeletal field, the degenerated and decreased muscle tissue has confused the clinical effects of various diseases [21, 22]. The regeneration and remobilization of degenerated and damaged muscle tissues have been a hot issue in the research [23]. Stem cells, owning to the myogenic differentiation, provide a possibility for the current issue. However, the specific key pathways and genes in the myogenesis of stem cells are still under mystic.

There stand various signaling pathways which were counted in the myogenic differentiate process of stem cells. Fu reported that PI3K pathway-related genes and proteins were upregulatedly expressed in the myogenic differentiation courses of mouse stem cells [10]. Meanwhile, upregulated p53 and actin signaling pathways were also proved to be responsible for the myogenesis of stem cells which were certified by Liu et al. [24] and Petschnik [24]. Except that, p38 signaling pathway and wnt pathway were both proved to be responsible for the process [25, 26]. As for the myogenic genes, MRFs, myoD, myoG, etc. [27, 28] were all reported as myogenic-related genes.

In the present study, a bioinformatics analysis was used to analyze the key pathways and hub genes in the myogenesis of stem cells based on 3 GEO databases. According to the analysis, a total of 824 DEGs were hunted out and applied for further GO and KEGG analyses to certify potential biological functions and pathways in myogenic differentiation. Except that, 111 genes from the top 3 clusters and 19 hub genes analyzed from the MCODE method were identified from the PPI network.

MyoD has been described as the decisive gene and component of diverting undifferentiated cells into myoblasts [29, 30]. Yamamoto [31] reported that the muscle satellite cells lacking myoD increased propensity for non-myogenic differentiation and concluded that myoD is a determinate factor that induced the stem cells to muscles. Meanwhile, several researches have shown that myoD play an important role in the myogenic process [32–34]. In Rudnicki [35] research, knock out of myoD and myf5 results in the prevention of formation of the skeletal muscle in the embryo period. The study results revealed that myoD and myof5 were determined genes in the origination of muscle cells. In the present bioinformatic analysis, myoD showed key effects in the myogenesis. In GO analysis of upregulated genes, myoD showed significance in positive regulation of myoblast differentiation in BP, transcription factor complex in CC and chromatin binding, transcription factor binding, and transcription factor activity in MF. Meanwhile, the GSEA analysis revealed that moyD were both core enriched elements in myogenesis and muscle cell development in the three GEO databases. The unit results from our analysis revealed that myoD can be one of the hub genes in the myogenic differentiating process. From the laboratory experiments, RT-PCR results revealed that myoD were exactly significantly upregulated in myogenic induced stem cells.

Also, we performed the KEGG analysis to trace out the exact relevant pathways in the myogenic differentiation not only in the DEGs, but also based on the intensive module analysis from the PPI network. From the DEGs, the activation of PI3K, actin cytoskeleton regulation, and p53 signaling pathway were proved to be tightly associated with the myogenesis process. Meanwhile, the intensive analysis showed that actin cytoskeleton regulation pathway was also enriched. Mistriotis [36] recovered the myogenic differentiation potential by restoring the actin organization which revealed that actin is necessary in the myogenic differentiation. Anna [27] found myogenic differentiating abilities in glandular stem cells which own the actin expression. Actin have been a widely spread method to determine the myogenesis in various researches [37–39]. But few researches have devoted to determine the specific time point for the expression of actin during the myogenic differentiation process. Based on our laboratory experiments, the expression of actin was exactly significantly upregulated in myogenic induced stem cells.

The study still has several limitations. Firstly, the included GEO profiles were still not rich enough. Secondly, the specific gene regulations in different time points of differentiation were omitted in our study. We still need to conduct further validated experiments to prove our speculation in the future.

## Conclusion

Our study identified a series of DEGs in the myogenic differentiation process compared to undifferentiated stem cells. The 19 hub genes ASXL1, BOC, CENPH, DIMT1, ESRP1, GLDC, HOXD3, IGFBP5, JUN, MGST1, MRPS34, MSTN, MYOD1, MYOG, NBAS, PLS1, POLR3G, RNF144B, and UST were selected from the series bioinformatics analysis. From the further GO and KEGG analyses, the pathways’ own enriched genes were selected. Our analysis revealed the hub genes and key pathways in the myogenic differentiation process of stem cells.

## Supplementary Information

**Additional file 1: Figure 3b.** GO analysis and KEGG analysis based on upregulated genes. (E) Individual KEGG terms and their corresponding genes in each group. (F) Top BP terms and their corresponding genes in GO functional analysis. **Figure 3c.** GO analysis and KEGG analysis based on upregulated genes. (G) Top CC terms and their corresponding genes in GO functional analysis. (H) Top MF terms and their corresponding genes in GO functional analysis. **Figure 4b.** GO analysis and KEGG analysis based on downregulated genes. (E) Individual KEGG terms and their corresponding genes in each group. (F) Top BP terms and their corresponding genes in GO functional analysis. **Figure 4c.** GO analysis and KEGG analysis based on downregulated genes. (G) Top CC terms and their corresponding genes in GO functional analysis. (H) Top MF terms and their corresponding genes in GO functional analysis. **Figure 5b.** Individual KEGG terms based on DEGs and their corresponding genes in each group.

## Data Availability

The data used and analyzed during the current study are available from the corresponding author on reasonable request.
